# Patient satisfaction in one-stage immediate breast reconstruction after mastectomy: A multi-center comparative patient evaluation of prosthesis, LDMF, and TRAM techniques

**DOI:** 10.1097/MD.0000000000019991

**Published:** 2020-05-29

**Authors:** Chuqi Lei, Lijie Xu, Feng Xu, Jie Li, Hongchuan Jiang, Shan Guan, Xiang Wang, Bing Wen, Jinfeng Li, Xiru Li, Cuizhi Geng, Jian Yin

**Affiliations:** a Department of Breast Surgery; b Department of General Surgery, Beijing Chao-Yang Hospital; c Department of General Surgery, Beijing Tongren Hospital; d Department of Breast Surgery, Cancer Hospital, Chinese Academy of Medical Sciences; e Department of Plastic and Reconstructive Surgery, The First Hospital of Peking University; f Department of Breast Center, Peking University Cancer Hospital; g Department of General Surgery, General Hospital of People's Liberation Army, Beijing; h Breast Center, The Fourth Hospital of Hebei Medical University, Shijiazhuang, Hebei; i Department of Breast Surgery, Tianjin Medical University Cancer Institute and Hospital, Tianjin, China.

**Keywords:** aesthetic satisfaction, general satisfaction, prosthesis, LDMF, TRAM

## Abstract

To analyze patient satisfaction and the predictive factors characterizing three types of one-stage immediate breast reconstruction (IBR) after mastectomy, including prosthesis, latissimus dorsi myocutaneous flap (LDMF), transverse rectus abdominis myocutaneous (TRAM) flap techniques.

Data were collected via face-to-face or telephone interviews from eight breast centers in China from January 2012 to December 2016. A standardized questionnaire that evaluated the general satisfaction and aesthetic satisfaction was sent to patients who had undergone IBR. Logistic regression analysis was performed to identify risk factors associated with patient satisfaction among the three types of breast reconstruction.

A total of 412 questionnaires were sent out, and 309 copies were collected including 226 prosthesis, 46 LDMF, and 37 pedicle TRAM reconstruction. Logistic regression analysis showed that general satisfaction and aesthetic satisfaction were significantly correlated with radiotherapy (*P* < .001, *P* = .018), respectively. Besides, the aesthetic satisfaction was also associated with nipple-areola complex (NAC) preservation (*P* < .001).

Our multi-center study identified factors of higher patient satisfaction, like NAC preservation and absence of radiotherapy, in order to help breast surgeons make better decisions about individualized reconstruction plan.

## 1 Introduction

As the population of patients with breast cancer has increased dramatically, they are in need of a personalized treatment which not only makes it possible to save their lives but also improves their quality of life.^[1]^ One-stage immediate breast reconstruction (IBR) has been demonstrated to be a reliable method to prevent the patient from body disturbances after mastectomy without affecting oncology safety.^[2]^ Thus, the demand for IBR has gradually increased over the last two decades, especially in China.^[3]^ Patients are starting to pay more attention to their cosmetic outcomes, so that their satisfaction about the operation plays a indispensable role in choosing the right treatment for surgeons. To date, a group of studies focusing on the patients’ satisfaction after the breast reconstruction surgery showed that age, BMI index and different reconstruction methods might be the important factors affecting patient satisfaction directly.^[4–6]^ However, the data support for one-stage IBR is still a deficiency. Thus, a multicenter large-scale study is needed. The present study, collecting data from eight breast centers in different Chinese cities, aims at evaluating the postoperative satisfaction of the patients with IBR and identifying the major factors affecting patient satisfaction most. Three main types of reconstruction methods were involved in this study: prosthetic implantation reconstruction, latissimus dorsi myocutaneous (LDM) flap reconstruction and pedicle transverse rectus abdominis musculocutaneous (TRAM) flap reconstruction. The conclusion of this study may provide surgeons and patients with validated information to choose reconstructive procedures more appropriately.

## 2 Methods

### 2.1 Patient population

The participants diagnosed as breast cancer, who underwent one-stage IBR after mastectomy from January 2012 to December 2016, were selected from eight breast centers at different grade III hospitals in China. A total of 412 patients from all eight hospitals were involved and agreed to participate in the study, of which 309 patients completed the questionnaires. A small number (n = 33) of randomly selected questionnaires were repeated to estimate reliability of the questionnaire. The repeat interviews were performed 2 months after the main study. Non-participants (n = 37) were mainly out of their own wishes and other patients (n = 66) were lost to follow-up. Among them, 226 patients chose the prosthetic implantation reconstruction, while 46 used LDM flap and 37 received TRAM flap. These patients were invited by face-to-face or telephone follow-up to complete the questionnaires. The selected participants signed written informed consent. This study was approved by the Ethics Review Board of Affiliated Beijing Chao-Yang Hospital, Capital Medical University.

### 2.2 Questionnaire and data collection

The work has been reported in line with the Standards for Reporting Qualitative Research (SRQR).^[7]^ Our research was registered at http://www.researchregistry.com (UIN: researchregistry4872). 80% of the interviewers have medical doctorates and hold administrative positions in clinical departments. The IBR surgery was performed by well-trained breast surgeons from eight breast centers. The basic information and surgery records of patients were collected through the electronic medical record system. All data were coded and processed anonymously to ensure the patients’ privacy to be well protected and confidential.

Satisfaction was assessed by a questionnaire on the basis of the Michigan Breast Reconstruction Outcomes Study, including general satisfaction and aesthetic satisfaction.^[8]^ The questionnaire was translated and back translated from English to Chinese accurately. As can be seen from Table 1, the questionnaire was comprised of seven questions which were divided into 2 parts. Part 1 including 5 questions was designed to assess general satisfaction, while other two questions in part 2 were designed for aesthetic satisfaction. According to Likert classification method, patient response to the questions was categorized by a five-point scale ranging from dissatisfaction to satisfaction. The results were separated into two parts: scores of 4 or 5 were considered as “satisfied” and those scores below 4 were considered as “dissatisfied.”

**Table 1:**
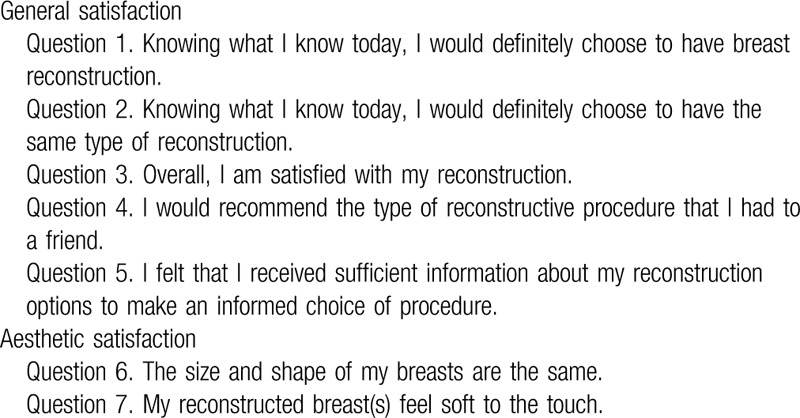
Satisfaction questions.

Apart from this, the original data involved sociodemographic, oncological, and clinicopathological factors, including age, BMI index, education level, smoking, chemotherapy, radiotherapy, preservation of nipple areola complex (NAC), tumor size (<2 cm, 2–5 cm, >5 cm), tumor type (ductal carcinoma in situ, invasive carcinoma), tumor stage (0 + I, II), and ER/PR/HER-2 status. The datasets generated during the present study are not publicly available, but are available from the corresponding authors on reasonable request.

### 2.3 Statistical analysis

A Student's *t* test was used to compare the continuous variables such as age and BMI index which were described by mean and standard deviation, whereas a Chi-square test was utilized for comparing the categorical variables. Summaries of clinicopathological characteristics among the three main reconstructive techniques were performed using descriptive statistics. Univariate analyses of factors associated with general satisfaction and aesthetic satisfaction were performed before multivariable logistic regression analysis. Then, a multiple logistic regression analysis was performed by setting satisfaction as the dependent variable, adopting the backward stepwise method. Adjusted odds ratios (OR), 95% confidence intervals (95%CI) and p values were derived from the regression analysis. All analyses were performed with SPSS 17.0 (Chicago, IL) and the statistical significance was considered as *P*-value < .05.

## 3 Results

### 3.1 Socio-demographic characteristics

A total of 412 questionnaires were sent out, and 309 copies were collected, with the responses rate at 75%. Among them, 226 patients underwent prosthetic reconstruction, while 46 patients received LDM flap, and 37 chose TRAM flap reconstruction. Patients’ characteristics based on different reconstruction methods were shown in Table 2. We found that 85% of the patients with breast cancer who chose the prosthetic reconstruction were younger than 45 years old, of which the population was far more than the LDMF and TRAM group, at 30.4% and 21.6%, respectively (*P* < .001). At the same time, the proportion of patients from the prosthetic group with BMI <28 kg/m^2^ was the highest (74.8%), while that of the LDMF and TRAM groups were less, with 54.3% and 40.5%, respectively (*P* < .001). It is noticeable that there were no significant differences among the three reconstruction methods in terms of educational status (*P* = .469), smoking (*P* = .055), postoperative chemotherapy (*P* = .540), radiotherapy (*P* = .830), NAC preservation (*P* = .327), tumor size (*P* = .751), pathological findings (*P* = .263), clinical stages (*P* = .481), and ER/PR/Her-2 status.

**Table 2:**
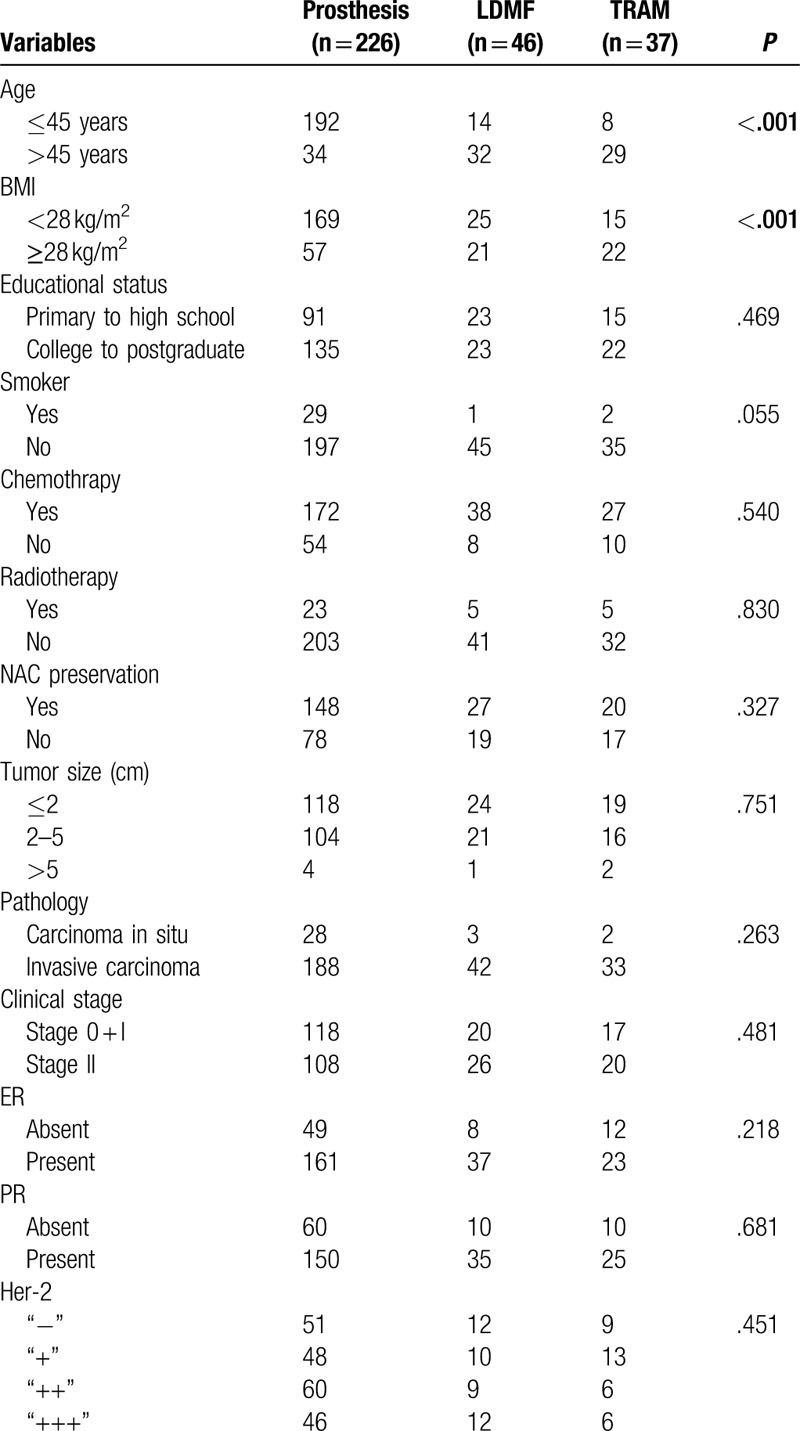
Sociodemographic and clinicopathological variables were compared for breast cancer patients based on reconstruction type.

### 3.2 Satisfaction assessment

According to the original data, the general and aesthetic satisfaction were evaluated. Multiple logistic regression analysis revealed (Table 3) that general satisfaction (OR = 0.253, 95%CI 0.136–0.470, *P* < .001) and aesthetic satisfaction (OR = 0.483, 95%CI 0.264–0.882, *P* = .018) were significantly correlated with radiotherapy, respectively. Apart from radiotherapy, the aesthetic satisfaction was also associated with nipple-areola complex (NAC) preservation (OR = 3.805, 95%CI 2.228–6.497, *P* < .001). Further analysis for the reconstruction types was performed and the result showed that there was no substantial difference in general and aesthetic satisfaction between the prosthetic group and the autologous group.

**Table 3:**
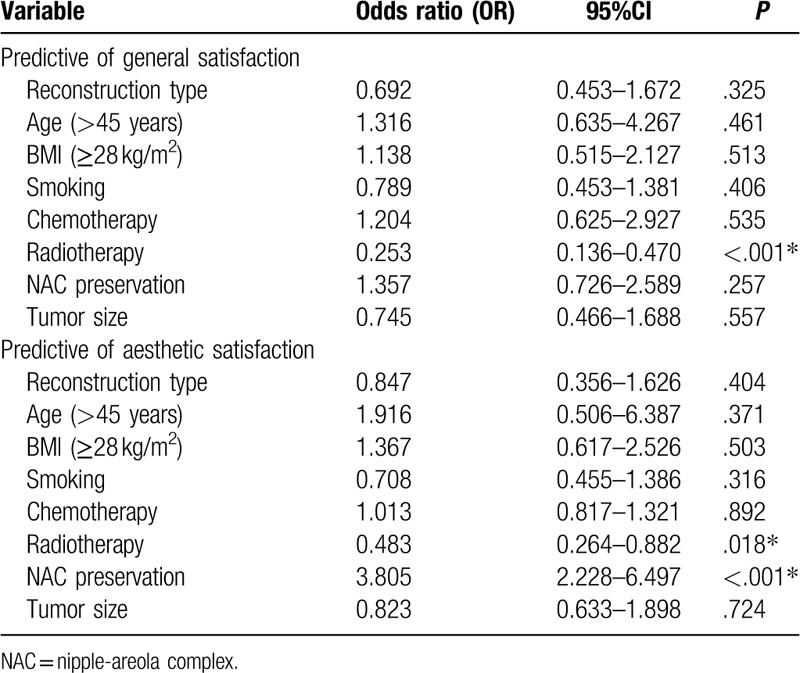
Predictors of general satisfaction and aesthetic satisfaction with breast reconstruction.

## 4 Discussion

The IBR after mastectomy has been widely carried out since women started to concern more about their physical aesthetics. More attention should be paid to the life quality of patients with breast cancer from clinical surgeons and patients themselves. Since Alderman brought forward the Michigan Breast Reconstruction Outcomes Study to the world in 2000, evaluating patient satisfaction after reconstruction has become not only a useful tool to improve quality of life, but also an effective way to appraise the effectiveness of reconstruction surgery. The body of literature^[9–11]^ about patient satisfaction after breast reconstruction has increased, whereas the reports comparing different types of one-stage IBR methods are still a deficiency. This is the reason why a multicenter large-scale study is needed.^[12]^ The present study compared the satisfaction among three types of IBR techniques including prosthesis implantation, LDMF and TRAM flap reconstruction with a multi-center large sample size and investigated the predictive factors that might affect the satisfaction.

In our study, the participants had similar educational background, family income, postoperative treatment (chemotherapy and radiotherapy), and other oncological factors (tumor size, location, pathological typing, and ER/PR/Her-2). In terms of the age and BMI index, there were significant differences existing among the three groups. Furthermore, most of the younger patients with lower BMI index were selected for the prosthetic implant reconstruction, while those who chose autologous reconstruction were generally older and had higher BMI index. The results are consistent with the previous study.^[13]^ In addition, compare to LDMF, the patients with TRAM technique were older and had higher BMI. The results of certain investigators were similar with us and they believed that older and obese patients were more likely to have enough abdominal tissue for TRAM flap reconstruction.^[14]^ Besides, younger patients were more likely to suffer from abdominal scarring physically, such as cesarean section surgery.

When it comes to satisfaction survey, the present study assessed the general and aesthetic satisfaction of 309 patients with IBR. The result indicated that different reconstruction methods could not affect patient satisfaction. Moreover, there was no significant difference in the satisfaction rate between TRAM and LDMF. Our findings were supported by the single-institute research of Fudan University Cancer Hospital.^[15]^ However, a study from Harvard medical school somehow stood in contradiction to ours. The Harvard researchers investigated 87 patients with tissue expander/implant, 116 with latissimus dorsi and 119 with pedicled TRAM and reported that patients with pedicle TRAM flap were the most satisfied. Similarly, Fracon^[16]^ also performed the cross-sectional study showing that autologous breast reconstruction had higher patient satisfaction than implant breast reconstruction. This inconsistency could be explained by the different population of the participants. To a certain extent, most of patients from China might have lower BMI index and smaller size of breast than the patients from America.

As for radiotherapy, it could effectively reduce the risk of local-regional recurrence (LRR) of patients with breast cancer.^[17]^ For any adjunctive therapy, there is a compromise between effect and side-effect, so as radiotherapy. The conclusions about the impact of postoperative radiotherapy on patient satisfaction were diverse. For example, Yueh^[14]^ did not find any radiational effect on patient satisfaction among the autologous and implant groups although they did not recommend this therapy after reconstruction. However, our result stood inconsistent with Yueh's study as we found that radiotherapy had greater impact on reducing the satisfaction aesthetically. Similarly, Albornoz^[17]^ also identified radiotherapy as a negative predictor for the satisfaction in patients with implant reconstruction. The difference might be explained by using the different questionnaires and the lower radiation rate in our study. It was suggested that radiotherapy had greater impact on breast shape recovery and life quality of patients after surgery. Furthermore, a large proportion of patients are dissatisfied with the size, shape and touch of the breast after radiotherapy because of the atrophy of subcutaneous fat, latissimus dorsi, pectoralis, and other tissue dysfunction caused by radiation.^[18]^ Therefore, the researchers had reason to believe that radiotherapy affected both general satisfaction and aesthetic satisfaction as a result of its related side effects.

As we all know, nipple-sparing mastectomy has been proved to be an oncological safe technique. According to previous studies, the NAC preservation had positive effect not only on patient body image but also on their sex lives.^[19]^ Didier^[10]^ built a new questionnaire to investigate patient satisfaction with or without NAC conservation. The collected data reported that patients with NAC preservation had minor aesthetic complains about patient satisfaction, body image and psychological adjustment. The result was consistent with the finding of Levy,^[11]^ who reported 18 patients with NAC conservation and identified a high score of satisfaction. In the present study, we found that all of the three reconstruction types had high rates of NAC preservation which set an agreement with Yueh^[20]^ who put forward a 90% satisfaction rate. Additionally, patients who conserved NAC had significantly higher aesthetic satisfaction than those who did not. The result further confirmed the benefit provided by NAC preservation and suggested surgeons to preserve NAC for patients as much as possible.

Admittedly, there are still some defects in this study. First, the surgical methods are not comprehensive enough. Except for LDMF and TRAM flap reconstruction, deep inferior epigastric perforator (DIEP) flap reconstruction and prosthesis combined with autologous reconstruction are also commonly used in clinic. Secondly, the population of the autogenous reconstruction group is relatively small, and the data needs to be further expanded. Thirdly, in addition to the Michigan scale, there are many other scales such as BREAST-Q,^[21]^ BCTOS that have ability to assess the patient satisfaction. Only one scale is not enough to assess patient satisfaction exhaustively. Fourthly, other factors that might affect patient satisfaction are not discussed in this study, such as different surgery timing, hospital costs and the impact of unilateral or bilateral surgery on patients. Further researches are needed for in-depth discussion.

To sum up, based on the multi-center data, the present study explored the satisfaction of patients who has undergone three different reconstructions, and analyzed the important factors affecting the satisfaction of patients. It was found that general satisfaction and aesthetic satisfaction were significantly correlated with radiotherapy. In addition, nipple-areola complex preservation was also an important factor affecting aesthetic satisfaction.

## References

[1] Platt J Zhong T. Patient-centered breast reconstruction based on health-related quality-of-life evidence. Clin Plastic Surg 2018;45:137–43. 10.1016/j.cps.2017.08.011 29080656

[2] Lee CN Pignone MP Deal AM. Accuracy of predictions of patients with breast cancer of future well-being after immediate breast reconstruction. JAMA Surg 2018;153:e176112. 29417143 10.1001/jamasurg.2017.6112 PMC5875341

[3] Jia-jian C Nai-si H Jing-yan X. Current status of breast reconstruction in Southern China: a 15 year, single institutional experience of 20,551 breast cancer patients. Medicine 2015;94:e1399. 26313786 10.1097/MD.0000000000001399 PMC4602900

[4] Alderman AK Wilkins EG Kim HM. Complications in postmastectomy breast reconstruction: two-year results of the Michigan Breast Reconstruction Outcome Study. Plastic Reconstruct Surg 2002;109:2265–74. 10.1097/00006534-200206000-00015 12045548

[5] Vyas RM Dickinson BP Fastekjian JH. Risk factors for abdominal donor-site morbidity in free flap breast reconstruction. Plastic Reconstruct Surg 2008;121:1519–26. 10.1097/PRS.0b013e31816b1458 18453973

[6] Colakoglu S Khansa I Curtis MS. Impact of complications on patient satisfaction in breast reconstruction. Plastic Reconstruct Surg 2011;127:1428–36. 10.1097/PRS.0b013e318208d0d4 21460651

[7] O’Brien BC Harris IB Beckman TJ. Standards for reporting qualitative research: a synthesis of recommendations. Acad Med 2014;89:1245–51. 24979285 10.1097/ACM.0000000000000388

[8] Alderman AK Wilkins EG Lowery JC. Determinants of patient satisfaction in postmastectomy breast reconstruction. Plastic Reconstruct Surg 2000;106:769–76. 10.1097/00006534-200009040-00003 11007387

[9] Murray CD Turner A Rehan C. Satisfaction following immediate breast reconstruction: experiences in the early post-operative stage. Br J Health Psychol 2015;20:579–93. 24946693 10.1111/bjhp.12112

[10] Didier F Radice D Gandini S. Does nipple preservation in mastectomy improve satisfaction with cosmetic results, psychological adjustment, body image and sexuality? Breast Cancer Res Treat 2009;118:623–33. 19003526 10.1007/s10549-008-0238-4

[11] Levy J Bosc R Warren N. Nipple-sparing mastectomy and immediate breast reconstruction with a deep inferior epigastric perforator flap: a study of patient satisfaction. Ann Plastic Surg 2018;80:639–43. 10.1097/SAP.0000000000001404 29664829

[12] Levine SM Levine A Raghubir J. A 10-year review of breast reconstruction in a university-based public hospital. Ann Plastic Surg 2012;69:376–9. 10.1097/SAP.0b013e31824b26d2 22868309

[13] Mioton LM Smetona JT Hanwright PJ. Comparing thirty-day outcomes in prosthetic and autologous breast reconstruction: a multivariate analysis of 13,082 patients? J Plastic Reconstruct Aesthetic Surg 2013;66:917–25. 10.1016/j.bjps.2013.03.009 23562485

[14] Yueh JH Slavin SA Adesiyun T. Patient satisfaction in postmastectomy breast reconstruction: a comparative evaluation of DIEP, TRAM, latissimus flap, and implant techniques. Plastic Reconstruct Surg 2010;125:1585–95. 10.1097/PRS.0b013e3181cb6351 20517080

[15] Yang B Li L Yan W. The type of breast reconstruction may not influence patient satisfaction in the Chinese population: a single institutional experience. PLoS One 2015;10:e0142900. 26562294 10.1371/journal.pone.0142900 PMC4642975

[16] Fracon S, Renzi N, Manara M, et al. Patient satisfaction after breast reconstruction: implants vs. autologous tissues. *Acta Chir Plast* 2018;59:120-128. 29651851

[17] Albornoz CR Matros E McCarthy CM. Implant breast reconstruction and radiation: a multicenter analysis of long-term health-related quality of life and satisfaction. Ann Surg Oncol 2014;21:2159–64. 24740825 10.1245/s10434-014-3483-2

[18] Cordeiro PG McCarthy CM. A single surgeon's 12-year experience with tissue expander/implant breast reconstruction: part II. An analysis of long-term complications, aesthetic outcomes, and patient satisfaction. Plastic Reconstruct Surg 2006;118:832–9. 10.1097/01.prs.0000232397.14818.0e 16980843

[19] Kadoch V Bodin F Himy S. Latissimus dorsi free flap for reconstruction of extensive full-thickness abdominal wall defect. A case of desmoid tumor. J Visceral Surg 2010;147:e45–8. 10.1016/j.jviscsurg.2010.05.005 PMC2924994 20692637

[20] Yueh JH Houlihan MJ Slavin SA. Nipple-sparing mastectomy: evaluation of patient satisfaction, aesthetic results, and sensation. Ann Plastic Surg 2009;62:586–90. 10.1097/SAP.0b013e31819fb1ac 19387167

[21] Mundy LR Homa K Klassen AF. Breast cancer and reconstruction: normative data for interpreting the BREAST-Q. Plastic Reconstructive Surg 2017;139 (5):1046e–55e. 10.1097/PRS.0000000000003241 PMC5713639 28445351

